# Linear Ubiquitination of Hemocyanin Mediated by LUBEL Regulates Innate Immunity in *Penaeus vannamei*

**DOI:** 10.3390/ijms26115110

**Published:** 2025-05-26

**Authors:** Xiaojun Zhang, Hanfeng Zhang, Yueling Zhang, Zhongyang Lin

**Affiliations:** Guangdong Provincial Key Laboratory of Marine Biotechnology, Institute of Marine Sciences, Shantou University, Shantou 515063, China; 17medxjzhang@stu.edu.cn (X.Z.); 20hfzhang@alumni.stu.edu.cn (H.Z.)

**Keywords:** hemocyanin, ubiquitination, *Penaeus vannamei*

## Abstract

*Penaeus vannamei* hemocyanin (*Pv*HMC) exhibits multifunctional roles in immunity, often mediated by various post-translational modifications. While linear ubiquitination catalyzed by LUBAC in mammals regulates immune signaling, its role in crustacean immunity remains unclear. Here, we investigated the regulatory mechanism of *Pv*HMC linear ubiquitination mediated by an E3 ligase *Pv*LUBEL (a HOIP homolog), with emphasis on its role in shrimp immunity defending against diverse pathogens. We detected linear ubiquitination of *Pv*HMC in multiple tissues, including hemocytes and the hepatopancreas. During *Vibrio parahaemolyticus* infection, the expression of *Pv*LUBEL and the level of *Pv*HMC linear ubiquitination were suppressed, whereas infection by white spot syndrome virus (WSSV) led to their upregulation. Structural analyses revealed that *Pv*LUBEL, which shares a conserved RING-IBR-RING (RBR) domain with mammalian HOIP, serves as the catalytic subunit. Notably, inhibition of *Pv*LUBEL via HOIPIN-1 (a covalent inhibitor) or RNA interference (RNAi) significantly reduced *Pv*HMC linear ubiquitination, thereby increasing pathogen proliferation and decreasing host survival. These findings unveil a novel post-translational regulatory mechanism in which *Pv*LUBEL-mediated linear ubiquitination of *Pv*HMC underpins the shrimp immune response against aquaculture pathogens.

## 1. Introduction

Ubiquitin is a small protein consisting of 76 amino acids, weighting approximately 8.5 kDa, and is highly conserved in eukaryotic evolution [1]. The conjugation of ubiquitin to substrates is tightly regulated by ubiquitin-activating enzyme (E1), ubiquitin-conjugating enzyme (E2), and ubiquitin ligase (E3) [2,3]. In canonical ubiquitination, the Gly76 residue of ubiquitin is covalently attached to substrate protein lysine (Lys) residues through isopeptide bonds [4,5]. Additionally, multiple ubiquitin moieties can form polyubiquitin chains through seven lysine residues (Lys6, Lys11, Lys27, Lys29, Lys33, Lys48, and Lys63), thereby conferring different functional outcomes on the substrates [6]. Linear ubiquitination (M1-linked ubiquitination) is generated by the head-to-tail linkage of the Gly76 residue in one ubiquitin molecule to the N-terminal methionine (Met1) residue of another. This process is predominantly catalyzed by the Linear Ubiquitin Chain Assembly Complex (LUBAC), which comprises HOIL-1-interacting protein (HOIP), HOIL-1 homolog (HOIL-1), and SHANK-associated RH domain-interacting protein (SHARPIN) [7,8,9]. Notably, HOIP, an RBR (RING-between-RING) E3 ligase, is the only known enzyme capable of catalyzing the synthesis of linear ubiquitin chains [10].

Numerous studies have demonstrated that LUBAC-mediated linear ubiquitination plays a pivotal role in cellular signal transduction within the mammalian innate immune system [11]. This includes its involvement in the NF-κB signaling pathway, the RIG-I-dependent activation of type I interferon (IFN-I) production, and the NACHT, LRR, and PYD domains-containing protein 3 (NLRP3) inflammasome-mediated inflammatory response [12,13,14]. The formation of linear ubiquitin chains is indispensable for the regulation of antiviral immunity and inflammatory responses. Although linear ubiquitination has been extensively investigated in mammals, its role in invertebrates remains largely unexplored. Recently, an E3 ubiquitin ligase, LUBEL, has been identified as an orthologue of mammalian HOIP, and its mediated linear ubiquitination positively regulates the heat shock response in *Drosophila* [15]. It has been shown that LUBEL synthesizes linear ubiquitin chains de novo, a process critical for *Drosophila* survival following oral infection with Gram-negative bacteria. This process is also essential for the activation of Relish-dependent antimicrobial peptide gene expression and for pathogen clearance [16]. Furthermore, linear ubiquitination is essential for *Drosophila* survival under oxidative stress, driven by activation of the NF-κB transcription factor Relish via the Imd pathway [17].

Hemocyanin is a copper-containing respiratory protein commonly found in mollusks and arthropods [18]. In *Penaeus vannamei* (*P. vannamei*), hemocyanin (*Pv*HMC) comprises two subunits (approximately 77 kDa and 75 kDa) and is typically assembled in hexameric or dodecameric forms [19]. *Pv*HMC performs multiple physiological functions, including oxygen transport, osmotic regulation, and energy storage [20,21,22]. Additionally, accumulating evidence indicates that *Pv*HMC participates in direct and indirect immune responses, including phenoloxidase activity, agglutination, and antibacterial and antiviral defenses [23,24]. The functional diversity of *Pv*HMC is linked to various post-translational modifications, such as glycosylation, phosphorylation, acetylation, and ubiquitination [25,26,27]. These studies collectively suggest that *Pv*HMC and its post-translational modifications play vital roles in the innate immune defense of shrimp.

*P. vannamei* is the most widely cultured shrimp species, favored for its short production cycle, high stocking density, and tolerance to low salinity [28]. However, monocultural and intensive aquaculture practices have led to frequent disease outbreaks [29,30]. Statistics indicated that viral pathogens, especially the white spot syndrome virus (WSSV), cause about 60% of losses [31]. Additionally, bacterial pathogens like *Vibrio parahaemolyticus* (*V. parahaemolyticus*) contribute to 20% of losses, causing acute hepatopancreatic necrosis disease (AHPND) [32]. This disease can lead to nearly 100% mortality in early-stage shrimp larvae, threatening the sustainable development of the shrimp aquaculture industry [33].

In this study, we characterized the linear ubiquitination of *Pv*HMC in shrimp and investigated its response to infections by *V. parahaemolyticus* and WSSV. Furthermore, we elucidated the mechanism underlying *Pv*HMC linear ubiquitination mediated by *Pv*LUBEL, and demonstrated that knockdown of *Pv*HMC linear ubiquitination, through inhibiting *Pv*LUBEL, increases shrimp susceptibility to pathogens and reduces shrimp survival. Our findings highlight the pivotal role of *Pv*HMC linear ubiquitination in modulating the innate immune response in shrimp.

## 2. Results

### 2.1. Identification and Tissue Distribution of Hemocyanin Linear Ubiquitination

To analyze the specificity of linear ubiquitinated antibodies, hemocyanin (*Pv*HMC) was enriched from shrimp hemocytes using rabbit anti-*Pv*HMC antibodies by immunoprecipitation (IP), with IgG as a control. Western blotting results show that rabbit anti-*Pv*HMC antibodies specifically enrich *Pv*HMC and detect clear linear ubiquitination signals, while no such signals are observed after IgG incubation (Figure S1A). This result indicates that the linear ubiquitinated antibodies are specific to *Pv*HMC.

**Figure 1:**
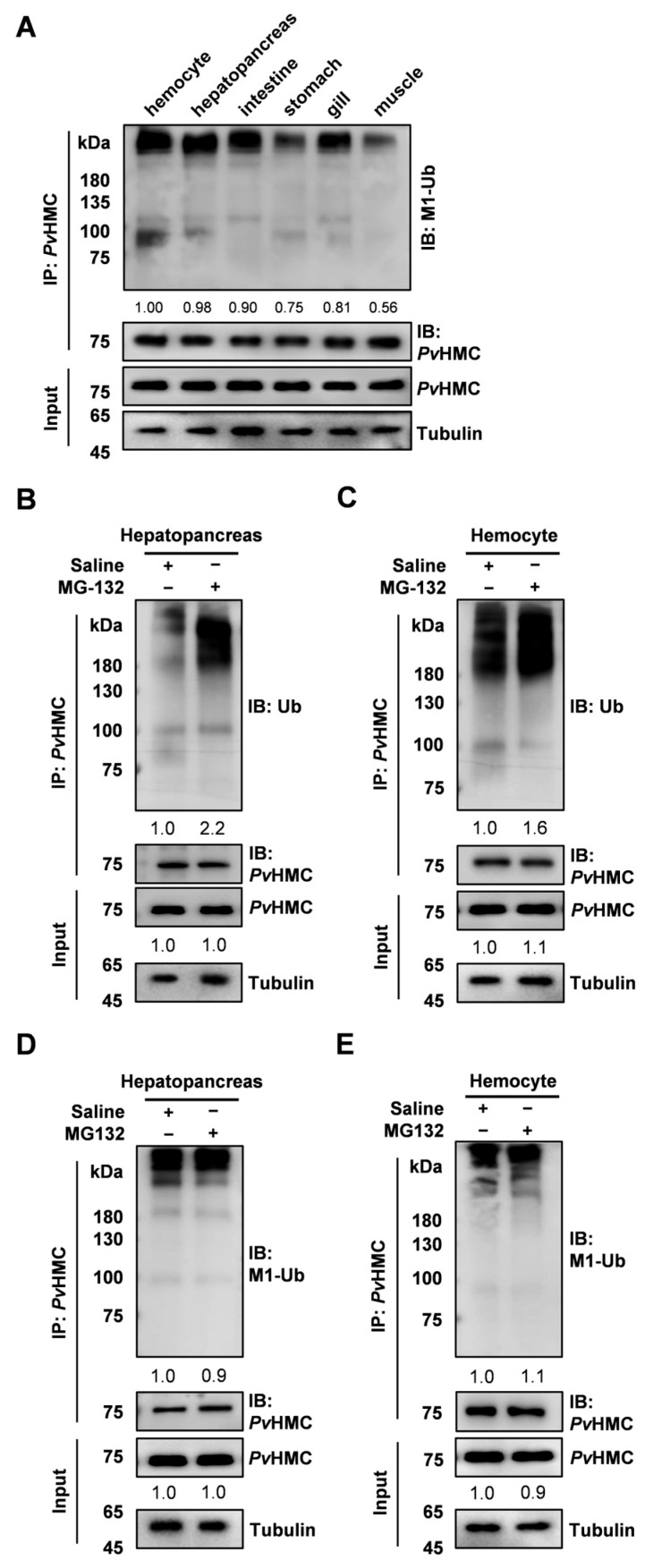
Identification of *Pv*HMC linear ubiquitination in various shrimp tissues. (**A**) Comparative analysis of *Pv*HMC linear ubiquitination in the hemocytes, hepatopancreas, intestine, stomach, gills, and muscle of healthy shrimp. (**B**) Western blotting analysis of *Pv*HMC total ubiquitination in the hepatopancreas after injection of 20 μM MG-132. (**C**) Western blotting analysis of *Pv*HMC total ubiquitination in the hemocytes following injection of 20 μM MG-132. (**D**) Western blotting analysis of *Pv*HMC linear ubiquitination in the hepatopancreas after injection of 20 μM MG-132. (**E**) Western blotting analysis of *Pv*HMC linear ubiquitination in the hemocytes following injection of 20 μM MG-132.

To investigate the tissue-specific distribution of *Pv*HMC linear ubiquitination, various shrimp tissues were collected and homogenized. *Pv*HMC was enriched using IP, and linear ubiquitination was detected by Western blotting (WB). The results showed diffuse signals above the molecular weight of *Pv*HMC (approximately 75 kDa), indicating that linear ubiquitination occurred across various tissues. Linear ubiquitination signals are most prominent in hemocytes, followed by the hepatopancreas (Figure 1A). However, no linear ubiquitination was detected in plasma, where E1, E2, and E3 enzymes are absent, either untreated or WSSV-stimulated (Figure S1B).

To evaluate the involvement of linear ubiquitination in the ubiquitin–proteasome pathway, the proteasome inhibitor MG-132 was injected. Although MG-132 markedly enhanced overall ubiquitination in hemocytes and the hepatopancreas (Figure 1B,C), its effect on linear ubiquitination levels was negligible, with almost no discernible change (Figure 1D,E). These findings suggest that *Pv*HMC linear ubiquitination does not participate in ubiquitin–proteasome-mediated degradation and may instead function in other signaling pathways.

### 2.2. Pathogen-Induced Changes in Linear Ubiquitination of Hemocyanin

**Figure 2:**
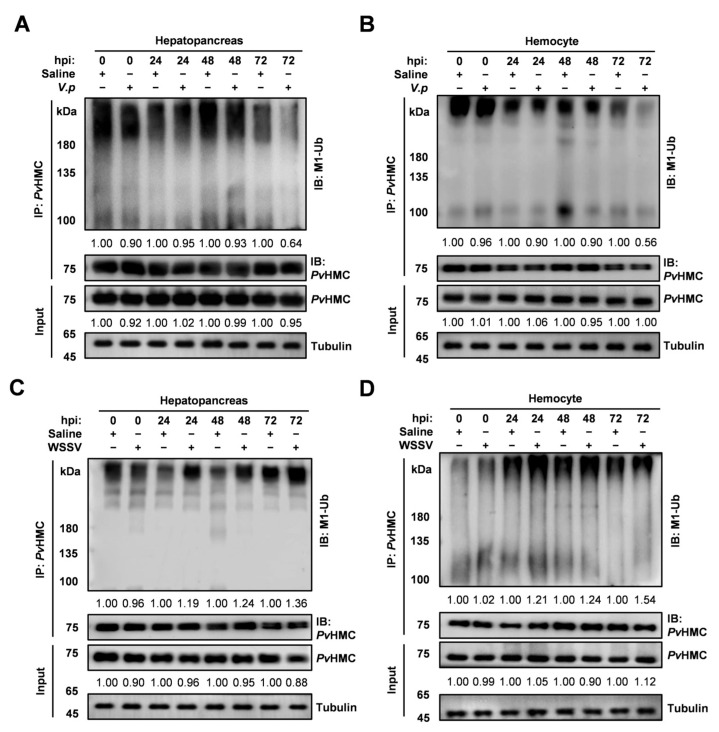
Changes in *Pv*HMC linear ubiquitination upon pathogen challenge. (**A**,**B**) Western blotting and relative grayscale analyses of *Pv*HMC linear ubiquitination and *Pv*HMC protein levels in the hepatopancreas (**A**) and hemocytes (**B**) at 0, 24, 48, and 72 h post-infection with *Vibrio parahaemolyticus* (*V.p*). (**C**,**D**) Western blotting and relative grayscale analyses of *Pv*HMC linear ubiquitination and *Pv*HMC protein levels in the hepatopancreas (**C**) and hemocytes (**D**) at 0, 24, 48, and 72 h post-infection with white spot syndrome virus (WSSV).

The hemocytes and hepatopancreas are key immune organs in shrimp, essential for resistance to pathogen invasion. Notably, *Pv*HMC linear ubiquitination is elevated in these organs. To investigate how linear ubiquitination of *Pv*HMC in the hemocytes and hepatopancreas responds to pathogen challenge, we infected shrimp with *Vibrio parahaemolyticus* (*V. parahaemolyticus*) or WSSV, using saline injection as a control. We found that during *V. parahaemolyticus* infection, *Pv*HMC linear ubiquitination in the hepatopancreas remained unchanged at 24 to 48 hpi but declined to 0.64-fold at 72 hpi (Figure 2A). Similarly, linear ubiquitination levels of *Pv*HMC remained stable at 24 to 48 hpi but decreased significantly at 72 hpi, reaching 0.56-fold at 72 hpi in the hemocytes (Figure 2B).

Conversely, WSSV infection led to a mild increase (1.19 to 1.24 at 24 to 48 hpi) in *Pv*HMC linear ubiquitination in the hepatopancreas, peaking at 1.36-fold at 72 hpi. Meanwhile, a decrease (0.12-fold) in the protein levels of *Pv*HMC was noted following WSSV infection (Figure 2C). In hemocytes, *Pv*HMC linear ubiquitination gradually increased (1.21 to 1.24-fold) from 24 to 48 hpi and rose sharply at 72 hpi, reaching 1.54-fold relative to controls. Additionally, a 0.12-fold increase in the protein levels of *Pv*HMC was observed following WSSV infection (Figure 2D). These results indicate that linear ubiquitination of *Pv*HMC responds to pathogen infection in a tissue-specific manner, potentially dictated by its regulatory ligases.

### 2.3. Identification and Functional Analysis of E3 Ubiquitin–Protein Ligase PvLUBEL

Linear Ubiquitin Chain Assembly Complex (LUBAC) is the sole E3 ligase known to catalyze linear ubiquitin chain formation in mammals [34]. HOIP is the core protein of LUBAC and contains the essential RING-IBR-RING (RBR) domain crucial for linear ubiquitin chain formation [35]. To uncover the E3 ligase mediating *Pv*HMC linear ubiquitination, we performed bioinformatics analysis and identified a *Penaeus vannamei* E3 ubiquitin–protein ligase (*Pv*LUBEL, Gene symbol: LOC113818292). This 3087-amino acid protein contains an RBR domain (2720–2936 aa), as confirmed by SMART (Figure S2A). Phylogenetic analysis revealed that *Penaeus* species formed a distinct clade (>90% homology), and *Pv*LUBEL shows >70% homology to *Homarus americanus*, *Procambarus clarkii*, and *Cherax quadricarinatus*. Other crustaceans (*Macrobrachium nipponense*, *Macrobrachium rosenbergii*, and *Scylla paramamosain*) cluster with arthropods, while insects comprise a separate branch (Figure S2B). Multiple sequence alignment demonstrated high conservation of the RBR domain among six species, underscoring its functional conservation in crustaceans (Figure S2C).

**Figure 3:**
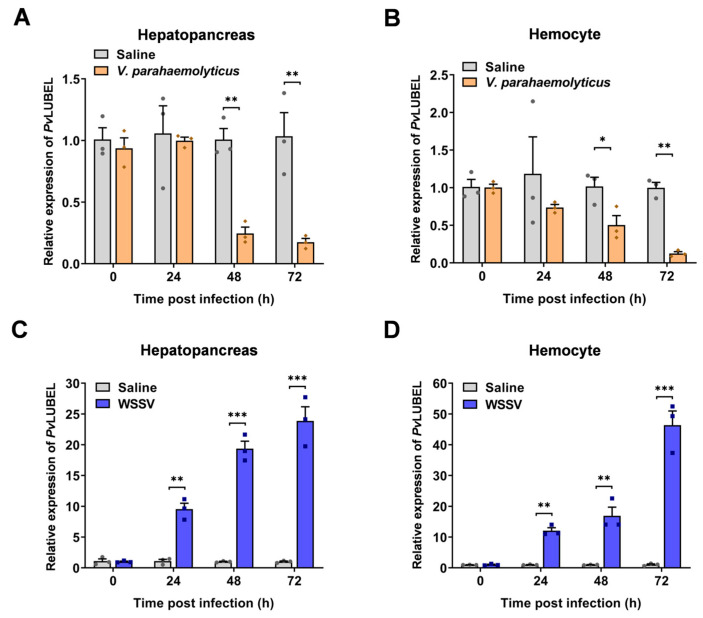
*Pv*LUBEL expression in response to pathogenic infection. (**A**,**B**) RT-qPCR analysis of *Pv*LUBEL expression in the hepatopancreas (**A**) and hemocytes (**B**) at 0, 24, 48, and 72 h post-*V. parahaemolyticus* infection. (**C**,**D**) RT-qPCR analysis of *Pv*LUBEL expression in the hepatopancreas (**C**) and hemocytes (**D**) at 0, 24, 48, and 72 h post-WSSV infection. *Pv*EF-1α was used as an internal control. Data are presented as mean ± SEM (n = 3). Statistical significance was determined by Student’s *t*-test: * *p* < 0.05, ** *p* < 0.01, *** *p* < 0.001.

We next examined the expression patterns of *PvLUBEL* by extracting total RNA from various tissues and performing RT-qPCR. The results showed that *PvLUBEL* is expressed at its highest levels in the hepatopancreas, followed by the hemocytes, stomach, and gills, with lower expression in the intestine and muscle (Figure S2D). Under *V. parahaemolyticus* challenge, *PvLUBEL* expression was markedly downregulated in both the hepatopancreas and hemocytes (Figure 3A,B). By contrast, WSSV infection significantly upregulated *PvLUBEL* expression in these tissues (Figure 3C,D). Taken together, these findings suggest that *PvLUBEL* is closely linked to shrimp defense against both bacterial and viral pathogens, implying an immune regulatory role during pathogen challenge.

### 2.4. HOIPIN-1 Suppresses PvHMC Linear Ubiquitination In Vivo

**Figure 4:**
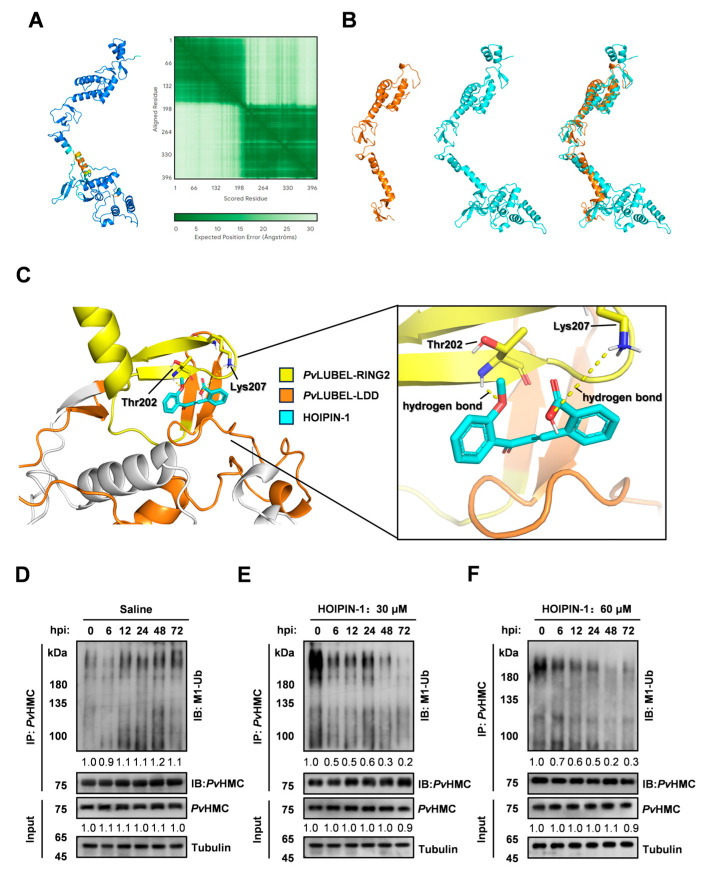
Molecular docking and in vivo analysis of HOIPIN-1′s interaction with and inhibition of *Pv*LUBEL. (**A**) Predicted structure of the *Pv*LUBEL-RBR domain generated by AlphaFold 3. (**B**) Superimposed three-dimensional structures of human HOIP (orange) and the *Pv*LUBEL (cyan) RBR domain, modeled using AutoDock and PyMOL. (**C**) Molecular docking results for HOIPIN-1 binding to the *Pv*LUBEL-RING2-LDD domain. Hydrogen bonds are indicated by dashed lines. Thr202 and Lys207 are crucial for interactions with HOIPIN-1. The left panel shows the overall view; the right panel shows a close-up view of the HOIPIN-1 binding site. (**D**–**F**) Western blotting and relative grayscale analyses of *Pv*HMC linear ubiquitination levels and protein levels were performed after injection of saline (**D**), and 30 μM (**E**) and 60 μM (**F**) HOIPIN-1 at 0, 6, 12, 24, 48, and 72 h post-injection.

To investigate the mechanism by which *Pv*HMC undergoes linear ubiquitination, we used AlphaFold to predict the spatial structure of the RING-IBR-RING-LDD domain of *Pv*LUBEL and compared it with human HOIP. The analysis revealed substantial structural similarity between *Pv*LUBEL and *Hs*HOIP, despite differences in spatial folding and amino acid length (Figure 4A,B), suggesting that *Pv*LUBEL may mediate linear ubiquitination in *P. vannamei*.

HOIPIN-1 (Synonyms: JTP-0819958), a selective LUBAC inhibitor, has been reported to block linear ubiquitin chain assembly, thereby suppressing NF-κB activation and phosphorylation of IKKα/β, p105, and p65 [36,37]. Molecular docking indicated that HOIPIN-1 binds to *Pv*LUBEL through hydrogen bonds formed between its benzoate carboxyl group and Lys207, as well as between its anisole ether bond and Thr202 in the RING2 domain (Figure 4C). These observations provide evidence that HOIPIN-1 targets *Pv*LUBEL’s RBR domain in a manner comparable to *Hs*HOIP.

To elucidate whether *Pv*LUBEL directly regulates *Pv*HMC linear ubiquitination, shrimp were injected with saline (control) or HOIPIN-1 (30 µM and 60 µM). After injecting 30 µM HOIPIN-1 at 6 hpi, the *Pv*HMC linear ubiquitination level decreased to 0.5-fold, and this inhibitory effect persisted up to 72 hpi (0.2-fold). The *Pv*HMC protein level showed a slight downregulation (0.9-fold) at 72 hpi. Following injection of 60 µM HOIPIN-1 at 6 hpi, the *Pv*HMC linear ubiquitination level decreased to 0.7-fold, and this inhibitory effect lasted up to 72 hpi (0.3-fold). The *Pv*HMC protein level also showed a slight downregulation (0.9-fold) at 72 hpi (Figure 4D–F). These findings demonstrate that *Pv*LUBEL activity is directly responsible for *Pv*HMC linear ubiquitination.

### 2.5. Inhibition of PvHMC Linear Ubiquitination Enhances Pathogen Proliferation

**Figure 5:**
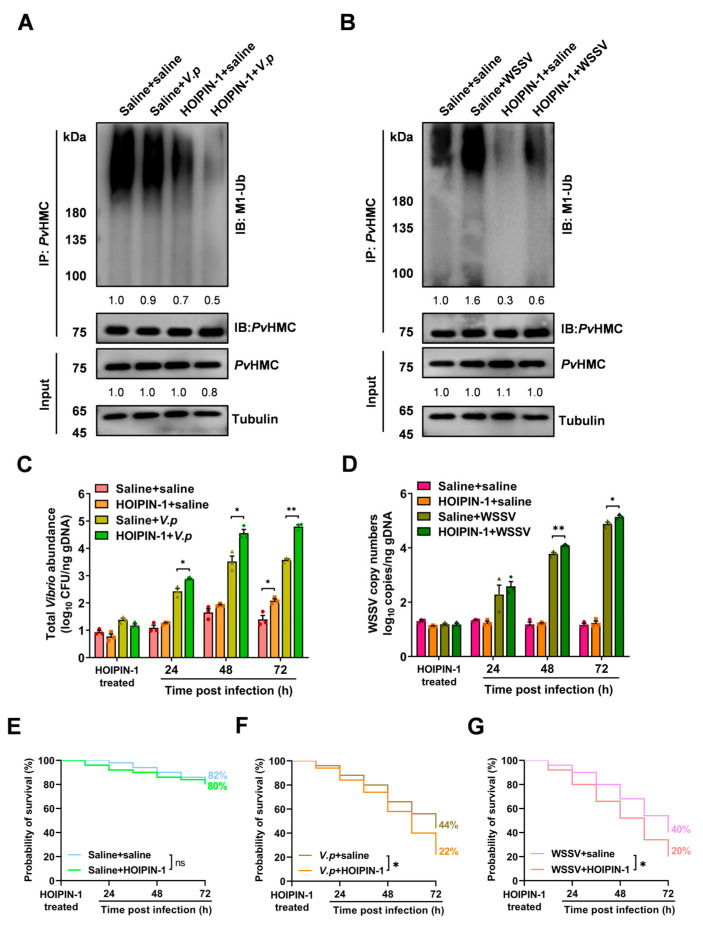
Effects of HOIPIN-1-mediated inhibition of *Pv*LUBEL on shrimp immunity. (**A**,**B**) Western blotting (WB) analysis of *Pv*HMC linear ubiquitination after inhibiting *Pv*LUBEL with HOIPIN-1 in *V. parahaemolyticus* (**A**) or WSSV (**B**) infection models. (**C**) RT-qPCR quantification of total *Vibrio* abundance at 24, 48, and 72 h post-*V. parahaemolyticus* infection in control and HOIPIN-1-treated shrimp. (**D**) RT-qPCR quantification of WSSV copy numbers at 24, 48, and 72 h post-infection in control and HOIPIN-1-treated shrimp. (**E**–**G**) Survival curves of shrimp after *Pv*LUBEL inhibition in uninfected controls (**E**), *V. parahaemolyticus* infection (**F**), or WSSV infection (**G**). Each group contained 50 animals. Statistical significance: * *p* < 0.05, ** *p* < 0.01, Student’s *t*-test. Data are shown as mean ± SEM from three independent experiments (n = 3).

To investigate the role of *Pv*LUBEL-mediated *Pv*HMC linear ubiquitination in immune defense and host resistance, we pretreated shrimp with HOIPIN-1 before challenging them with *V. parahaemolyticus* or WSSV. Western blotting analysis revealed that after *V. parahaemolyticus* challenge, *Pv*HMC linear ubiquitination levels in hemocytes decreased to 0.9-fold, compared with the control (saline + saline) group. Furthermore, the combination of HOIPIN-1 treatment and *V. parahaemolyticus* infection exacerbated this reduction (0.5-fold) (Figure 5A). In contrast, WSSV infection elevated linear ubiquitination signals to 1.6-fold, while the HOIPIN-1 + WSSV group (0.6-fold) still showed higher levels than the HOIPIN-1 + saline group (0.3-fold) (Figure 5B), suggesting that viruses may preserve linear ubiquitination through alternate pathways.

We next investigated the effect of HOIPIN-1 on pathogen proliferation at various time points in hemocytes. The results demonstrated that, after HOIPIN-1 treatment at 72 hpi, the total *Vibrio* abundance in the HOIPIN-1 group significantly increased to 2.1 log_10_ CFU/ng gDNA, which was 0.7-fold higher than that in the saline group (1.4 log_10_ CFU/ng gDNA) (*p* < 0.05). At 24, 48, and 72 hpi with *V. parahaemolyticus*, the total *Vibrio* abundance in the HOIPIN-1 + *V.p* group reached 2.8, 4.5, and 4.8 log_10_ CFU/ng gDNA, respectively, which were 0.45-, 1.0-, and 1.2-fold higher than that in the saline + *V.p* group (2.4, 3.5, and 3.6 log_10_ CFU/ng gDNA), significantly (Figure 5C). Similarly, the WSSV copy numbers in the HOIPIN-1 + WSSV group at 24, 48, and 72 hpi were 2.6, 4.1, and 5.1 log_10_ copies/ng gDNA, respectively, which were 0.3-, 0.3-, and 0.25-fold higher than those in the saline + WSSV group (2.3, 3.8, and 4.8 log_10_ copies/ng gDNA), significantly (Figure 5D). These findings suggest that inhibition of *Pv*HMC linear ubiquitination by HOIPIN-1 compromises the host’s ability to resist and clear pathogens.

Survival analysis showed that the HOIPIN-1-only treatment group (80%) was slightly reduced by 2% (not significant) under uninfected conditions, compared to the saline control group (82%) (Figure 5E). However, in the *V. parahaemolyticus* infection model, survival decreased significantly by 22% in the *V.p* + HOIPIN-1 group (22%) after 72 h, compared to the *V.p* + saline group (44%) (Figure 5F). Similarly, in the WSSV infection model, HOIPIN-1 treatment led to a 20% reduction in the WSSV + HOIPIN-1 group (20%), compared to the WSSV + saline group (40%) (Figure 5G). Collectively, these results indicate that inhibition of *Pv*HMC linear ubiquitination compromises host immune defense.

### 2.6. Impact of PvLUBEL Knockdown on Pathogen Proliferation and Shrimp Survival

**Figure 6:**
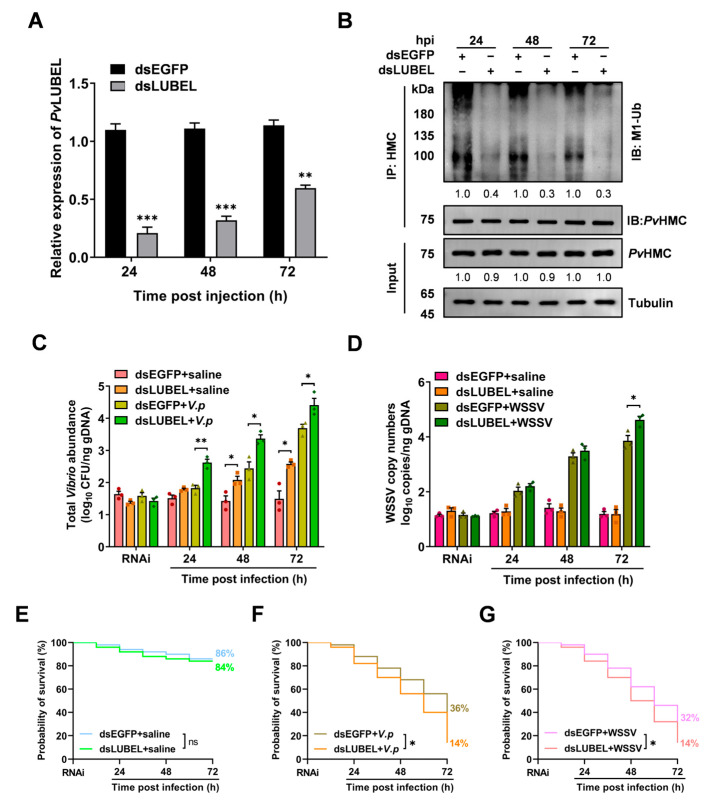
Impact of *Pv*LUBEL knockdown on shrimp immunity. (**A**) *Pv*LUBEL expression levels in vivo at 24, 48, and 72 h post-dsLUBEL injection, measured by RT-qPCR. (**B**) Western blotting (WB) analysis of *Pv*HMC linear ubiquitination at 24, 48, and 72 h post-dsLUBEL injection, with dsEGFP serving as a control. (**C**) Total *Vibrio* abundance after dsLUBEL treatment at 24, 48, and 72 h post-*V. parahaemolyticus* infection. (**D**) WSSV copy numbers after dsLUBEL treatment at 24, 48, and 72 h post-WSSV infection. (**E**–**G**) Survival curves of shrimp after *Pv*LUBEL knockdown in uninfected controls (**E**), *V. parahaemolyticus* infection (**F**), or WSSV infection (**G**). Each group contained 50 animals. Data are presented as mean ± SEM (n = 3). Statistical significance: * *p* < 0.05, ** *p* < 0.01, *** *p* < 0.001, Student’s *t*-test.

To further clarify the role of *Pv*LUBEL-mediated *Pv*HMC linear ubiquitination in shrimp anti-pathogen immunity, we suppressed *Pv*LUBEL expression using RNA interference (RNAi). RT-qPCR results indicated that 24, 48, and 72 h after dsLUBEL injection, *Pv*LUBEL expression decreased significantly by 79%, 68%, and 40%, respectively (Figure 6A). WB analysis confirmed a significant reduction in *Pv*HMC linear ubiquitination at these time points (Figure 6B). These findings underscore *Pv*LUBEL as a key regulator of *Pv*HMC linear ubiquitination.

We then examined the effect of dsLUBEL injection on pathogen proliferation in hemocytes. The results showed that, after dsLUBEL treatment at 48 and 72 hpi, the total *Vibrio* abundance in the dsLUBEL + saline group (2.1 and 2.6 log_10_ CFU/ng gDNA) was significantly increased, 0.7- and 1.1-fold higher, respectively, than that in the dsEGFP + saline group (1.4 and 1.5 log_10_ CFU/ng gDNA) under uninfected conditions. At 24, 48, and 72 hpi with *V. parahaemolyticus*, the total *Vibrio* abundance in the dsLUBEL + *V.p* group (2.6, 3.3, and 4.4 log_10_ CFU/ng gDNA) significantly increased by 0.8-, 0.9-, and 0.7-fold compared to the dsEGFP + *V.p* group (1.8, 2.4, and 3.7 log_10_ CFU/ng gDNA) (Figure 6C). Similarly, at 72 h post-WSSV infection, the WSSV copy numbers in the dsLUBEL + WSSV group (4.6 log_10_ copies/ng gDNA) increased by 0.8-fold compared to the dsEGFP + WSSV group (3.8 log_10_ copies/ng gDNA) (Figure 6D). These results suggest downregulation of *Pv*HMC linear ubiquitination leads to a reduction in the host’s resistance.

Survival analysis showed no significant change in the dsLUBEL + saline group (84%) compared to the dsEGFP + saline group (86%) under uninfected conditions (Figure 6E). However, in the *V. parahaemolyticus* infection model, the survival rate of the dsLUBEL + *V.p* group was 14% at 72 hpi, which was significantly lower than the 36% survival rate in the dsEGFP + *V.p* group (Figure 6F). In the WSSV infection model, the survival rate of the dsLUBEL + WSSV group decreased to 14%, a significant reduction of 18% compared to the 32% survival rate in the dsEGFP + WSSV group (Figure 6G).

Our findings demonstrate that *Pv*LUBEL-mediated linear ubiquitination of *Pv*HMC is crucial for shrimp immune defense against pathogens. Disruption of this process facilitates pathogen proliferation and significantly weakens the host’s ability to resist pathogen invasion and survive.

## 3. Discussion

*Pv*HMC plays a critical role in the innate immune system, contributing to diverse direct or indirect immune responses. Its functional diversity is closely associated with various post-translational modifications, as previously reported [38,39]. In this study, we focused on the role of *Pv*HMC linear ubiquitination in shrimp immunity. Our findings indicated that *Pv*HMC linear ubiquitination is widely distributed in shrimp tissues. Remarkably, in addition to the strong linear ubiquitination signals observed in hemocytes and the hepatopancreas, *Pv*HMC also exhibited linear ubiquitination in other immune-related organs such as the intestine and gills (Figure 1A). Both the hemocytes and hepatopancreas are key immune-related organs with high *Pv*HMC expression, possibly compensating for the lack of an adaptive immune system in crustaceans [40,41]. Therefore, the linear ubiquitination of *Pv*HMC expressed in these tissues likely holds significant immunological significance. Interestingly, no linear ubiquitination signal from *Pv*HMC was detected in plasma (Figure S1A), suggesting that this modification operates predominantly at the intracellular level. Linear ubiquitination, encompassing E1, E2, and E3 enzyme activities, is catalyzed by the LUBAC complex, which assembles linear ubiquitin chains [35]. However, the precise conditions needed for linear ubiquitination to occur in shrimp hemolymph remain elusive, and further research is warranted to elucidate its regulatory function in shrimp immunity.

Mammals often exhibit distinct immune responses to bacterial versus viral infections, whereas crustaceans tend to show comparable immunological responses [42]. Previous research has indicated that *Pv*HMC can be cleaved into antimicrobial peptides, such as *Pv*HMCS4, *Pv*HMCs27, and *Lv*HcL48 [23,43]. Nonetheless, when we applied the proteasome inhibitor MG-132, we observed an elevation in total *Pv*HMC ubiquitination in the hemocytes and hepatopancreas, whereas linear ubiquitination and overall protein levels remained unaffected (Figure 1B–E). This finding indicates that linear ubiquitination does not participate in *Pv*HMC degradation via the proteasomal pathway [44]. Generally, the ubiquitin–proteasome system regulates protein turnover, with K48-linked ubiquitination serving as the primary degradation signal [45]. In contrast, other types of ubiquitination are involved in cellular processes such as DNA repair and signal transduction [46]. We also examined dynamic changes in *Pv*HMC linear ubiquitination during pathogen infections. After *Vibrio parahaemolyticus* (*V. parahaemolyticus*) infection, *Pv*HMC linear ubiquitination decreased (Figure 2A,B), suggesting that *V. parahaemolyticus* may evade immune system detection by suppressing the *Pv*HMC immune response [47]. Conversely, after white spot syndrome virus (WSSV) infection, linear ubiquitination of *Pv*HMC was significantly upregulated (Figure 2C,D), suggesting a host mechanism to enhance immune interactions, activate immune pathways, and enhance pathogen clearance [48].

The ubiquitin machinery relies on E3 ligases to recognize substrates and regulate ubiquitination, thereby shaping various immune responses [49]. To clarify the distinct mechanisms governing *Pv*HMC linear ubiquitination during *V. parahaemolyticus* and WSSV infections, we investigated the relevant E3 ligases. In mammals, the LUBAC complex, which synthesizes linear ubiquitin chains, plays a pivotal role in immune regulation [50]. Here, we identified the *P. vannamei* ortholog of the LUBAC catalytic subunit HOIP, designated *Pv*LUBEL, whose RING-IBR-RING domain is highly conserved in crustaceans (Figure S2C). Phylogenetic analysis revealed that *Pv*LUBEL forms a distinct branch among crustaceans (Figure S2B), implying a conserved function of linear ubiquitination across different species. Structural domain assessments showed that *Pv*LUBEL’s RBR domain parallels that of mammalian *Hs*HOIP-RBR, reinforcing its key role in linear ubiquitin chain formation [35,51]. However, the absence of the PUB domain suggests that *Pv*LUBEL could form functional complexes through alternative assembly methods, possibly adapting to the demands of crustaceans’ open circulatory systems [52,53]. Moreover, RT-qPCR results showed that *V. parahaemolyticus* infection downregulated *Pv*LUBEL expression and correspondingly reduced *Pv*HMC linear ubiquitination (Figure 2A,B and Figure 3A,B), while WSSV infection upregulated *Pv*LUBEL expression and elevated *Pv*HMC linear ubiquitination (Figure 2C,D and Figure 3C,D). These findings highlight a direct relationship between *Pv*LUBEL levels and *Pv*HMC linear ubiquitination during pathogen stimulation. Notably, the hepatopancreas and hemocytes, which exhibit robust *Pv*LUBEL expression, participate actively in shrimp immunity, thereby reinforcing host defense via immune modulation.

LUBAC inhibitors, such as HOIPIN-1/8, are known to downregulate linear ubiquitination and suppress LUBAC-driven NF-κB activity in mammalian cells [37,54]. HOIPIN-1 binds covalently to the catalytic cysteine (Cys885) in the human HOIP-RING2 domain via a Michael addition reaction, blocking the RING-HECT hybrid mechanism [54]. Structural predictions indicate that the RBR domain of *Pv*LUBEL closely resembles that of *Hs*HOIP, differing by only one amino acid, suggesting that crustaceans and mammals may share analogous LUBAC active sites (Figure 4B). Molecular docking further showed that HOIPIN-1 interacts with the catalytic residues (Thr202 and Lys207) of the RING2 domain in *Pv*LUBEL through hydrogen bonds, mirroring its interaction with *Hs*HOIP (Figure 4C). Consequently, HOIPIN-1 emerged as a potential inhibitor of *Pv*LUBEL. In *vivo* assays supported this by revealing that injection of 30–60 μM HOIPIN-1 significantly suppressed *Pv*HMC linear ubiquitination in hemocytes for up to 72 h (Figure 4D–F). These data underscore *Pv*LUBEL*’*s pivotal role as an E3 enzyme responsible for *Pv*HMC linear ubiquitination.

It is therefore inferred that the core structure and function of LUBAC-mediated linear ubiquitination are highly conserved across arthropods and vertebrates, highlighting its importance in innate immune responses. To elucidate the protective role of *Pv*LUBEL-mediated *Pv*HMC linear ubiquitination against pathogen invasion, we employed HOIPIN-1 inhibitors and RNA interference (RNAi). Both dsLUBEL injection and HOIPIN-1 treatment consistently inhibited *Pv*HMC linear ubiquitination, sustaining their effects over extended periods. In infection models, *V. parahaemolyticus* challenge exacerbated the decline in *Pv*HMC linear ubiquitination and increased total *Vibrio* abundance (Figure 5A,C), suggesting that *V. parahaemolyticus* may further undermine host defense via a feedback mechanism [55]. By contrast, HOIPIN-1 exerted a relatively weaker inhibitory effect on WSSV infection, although linear ubiquitination remained elevated, and virus copy numbers rose slightly, indicating minimal influence on viral replication (Figure 5B,D). This suggests that additional E3 ligases or immune factors might also participate in regulating linear ubiquitination during viral infection [56]. Importantly, inhibition of *Pv*HMC linear ubiquitination led to significantly lower shrimp survival rates (Figure 5E–G and Figure 6E–G), correlating with higher *Vibrio* abundance and virus copies (Figure 6C,D), further confirming the critical role of *Pv*HMC linear ubiquitination in antimicrobial and antiviral immunity.

## 4. Materials and Methods

### 4.1. Penaeid Shrimp Sample Collection and Preparation

All animal experimental procedures were performed in accordance with the guidelines and approval of the Animal Research and Ethics Committee of Shantou University, Shantou, China (Approval Code: 202504002). The healthy *P. vannamei* shrimp, with an average weight of 8–10 g, were obtained from a local shrimp farm in Shantou, Guangdong Province, China (23.28° N, 116.69° E), and raised in laboratory tanks. The shrimps were cultured under continuous aeration in artificial seawater with 0.5% salinity at 25 °C, fed with commercial diets for 2 to 3 days, and acclimated to the laboratory conditions.

The sample collection methods of the hemolymph and hepatopancreas were described in previous studies [57]. In brief, hemolymph was extracted from the pericardial sinus using a sterile syringe, then mixed with 0.5 mL of cold anticoagulant buffer (450 mM NaCl, 10 mM KCl, 10 mM EDTA-Na_2_, and 10 mM HEPES, pH 7.0) in an Eppendorf tube (Eppendorf, Hamburg, Germany). The mixture was centrifuged at 800× *g* for 10 min at 4 °C to separate hemocytes and plasma. Hepatopancreatic samples were ground with a 150 μm steel sieve and homogenized in 1 mL of 10 mM PBS (pH 7.2) with 5 mM PMSF (Beyotime Biotechnology, Shanghai, China), then centrifuged at 100× *g* for 10 min at 4 °C to isolate hepatopancreatic cells. Shrimp tissues, including the intestines, stomach, gills, and muscle, were collected and instantly frozen with liquid nitrogen, then stored at −80 °C for further analysis.

### 4.2. Protein Extraction and Immunoprecipitation

The different tissues from shrimp were homogenized and lysed in immunoprecipitation (IP) buffer (25 mM Tris-HCl, pH 7.4, 150 mM NaCl, 1 mM EDTA, 1% NP-40, and 1% sodium deoxycholate)(Sangon Biotech, Shanghai, China) supplemented with protease inhibitor mixture (MedChemExpress, Monmouth Junction, NJ, USA), followed by centrifugation at 12,000× *g* for 15 min at 4 °C. The protein content of the supernatant was determined using a BCA assay kit (Beyotime Biotechnology, Shanghai, China).

For IP, Protein A/G Magnetic Beads (HY-K0202-1 mL; MedChemExpress, NJ, USA) were washed with PBST buffer (10 mM PBS, pH 7.4, and 0.5% Triton^TM^ X-100)(Merck, Darmstadt, Germany) and incubated with *Pv*HMC antibody (laboratory preparation) at 4 °C for 2 h. After magnetic separation, the beads were incubated with total protein lysate for 2 h. The complex was washed to eliminate nonspecifically bound proteins. SDS-PAGE loading buffer (10 mM Tris-HCl, pH 6.8, containing 2% glycerol, 5 g/L SDS, 2% 2-ME, and 0.01 g/L bromophenol blue, *w*/*v*) (Beyotime Biotechnology, Shanghai, China) was added, and the mixture was boiled for 5 min. The sample was separated using polyacrylamide gel electrophoresis, and subsequently analyzed by Western blotting.

### 4.3. RNA Interference and Pathogen Challenge

RNA interference was performed to investigate the function of *Pv*HMC linear ubiquitination. Double-strand RNA (dsRNA) targeting *Pv*LUBEL and EGFP (as a negative control) was synthesized using the HiScribe T7 Quick High Yield RNA Synthesis Kit (New England Biolabs, Ipswich, MA, USA). A 477 bp cDNA fragment of *Pv*LUBEL containing the T7 promoter sequence was used to generate the dsRNA (primers are provided in Supplemental Table S1). The dsRNAs were purified by isopropanol precipitation. Healthy shrimp were injected with 10 μg of dsLUBEL or dsEGFP. Hemolymph and hepatopancreas samples were collected at 24, 48, and 72 h post-dsRNA injection for RNA extraction, and *Pv*LUBEL expression was quantified by real-time quantitative PCR (RT-qPCR).

For the bacterial challenge, *V. parahaemolyticus* was cultured to an OD600 of ~0.4 and diluted based on a quantification standard curve. For the viral challenge, WSSV was propagated in crayfish, and WSSV particles were obtained from filtered hemolymph. Shrimp were injected with either 1 × 10^6^ CFU/mL *V. parahaemolyticus* or WSSV, with control shrimp receiving sterile saline. Hemocytes and hepatopancreatic samples were collected at various time points (0, 24, 48, and 72 h) for protein extraction. IP was performed as previously described, followed by Western blotting to assess the levels of ubiquitination, with tubulin as an internal control.

Survival rates were also recorded at different time points (0, 24, 48, and 72 h) after dsRNA injection and pathogenic infection. Each group consisted of 50 shrimp, with experiments conducted in triplicate for reproducibility.

### 4.4. Molecular Docking Simulation and Inhibitor Treatment

Receptor and ligand structure preparation: The RBR domain of *Homo sapiens* HOIP (*Hs*HOIP, gene name RNF31, AF-Q96EP0-F1-v4) was retrieved from the AlphaFold Protein Structure Database [58], while the *Penaeus vannamei* LUBEL (XP_069991990.1) sequence was obtained from the National Center for Biotechnology Information (NCBI) [59], then submitted to the Simple Modular Architecture Research Tool (SMART) for domain analysis [60]. The structure of HOIPIN-1 (External ID: HY-122881; PubChem SID: 461505538) was downloaded from PubChem (National Library of Medicine) [54]. The receptor structure was processed by removing water molecules, adding hydrogen atoms, and performing energy minimization. Molecular docking was carried out using AutoDock (version 4.2.6, Scripps Research, San Diego, CA, USA) [61], defining the receptor’s charge state and ligand docking region, with semi-flexible docking and 50 genetic algorithm (GA) runs. Binding affinity was evaluated based on docking scores, binding energies, and key interactions, including hydrogen bonds. Final models were refined through iterative optimization, and molecular graphics were visualized with PyMOL (version 3.0.3, Schrödinger, LLC, Palo Alto, CA, USA) [62].

### 4.5. Total RNA Extraction, cDNA Synthesis, and qRT-PCR

Total RNAs from different tissues were extracted using the RNAfast2000 Kit (Shanghai Feijie, Shanghai, China), in accordance with the manufacturer’s protocol. After liquid nitrogen grinding, column adsorption and purification, RNA was eluted and quantified by a Nanodrop One spectrophotometer (Thermo Scientific, Waltham, MA, USA). For cDNA synthesis, 1.0 μg of total RNA was utilized, using the TransScript One-Step gDNA Removal and cDNA Synthesis SuperMix Kit (TransGen Biotech, Beijing, China).

For qRT-PCR, the qPCR mixture contained 5 µL of 2× RealStar Green Power SYBR qPCR Mix (GenStar Biotech, Beijing, China), 1 µL of cDNA (10 ng/µL), 0.5 µL each of forward and reverse primers (5 µM), and Milli-Q water to a final volume of 10 µL. The qRT-PCR was performed in triplicate on a LightCycler 480II (384-well) system (Roche, Basel, Switzerland) with the following conditions: 55 °C for 10 min, initial denaturation at 95 °C for 5 min, followed by 40 cycles of 95 °C for 15 s and 60 °C for 30 s. Relative gene expression was calculated using the 2^−ΔΔCT^ method, with *Pv*EF-1α as the internal control. Primer sequences are listed in Table S1.

### 4.6. Western Blotting Analysis

Total protein lysates or IP samples were separated by 8–12% SDS-PAGE, then transferred to PVDF membranes (Millipore, Boston, MA, USA) and blocked with 5% skimmed milk in TBS-T buffer (20 mM Tris, 150 mM NaCl, and 0.1% Tween 20, pH 7.6) at room temperature for 1 h. After overnight incubation at 4 °C with specific antibodies (rabbit anti-M1-linked polyubiquitin Ab, 1:1000, ABclonal, Wuhan, China; rabbit anti-*Pv*HMC Ab, 1:5000, Abmart, Shanghai, China; and mouse anti-tubulin Ab, 1:3000, Merck, Darmstadt, Germany), the membrane was washed three times with TBS-T buffer, then incubated with HRP-conjugated anti-rabbit IgG (1:5000, Beyotime Biotechnology, Shanghai, China) for 1 h. Chemiluminescent detection was performed using an ECL reagent (Millipore, Boston, MA, USA), and images were captured with the GE Amersham Imager 600 system. Densitometric analysis of immunoreactivity was quantified using the ImageJ software (version 1.53q, NIH, Bethesda, MD, USA) [63].

### 4.7. Statistical Analysis

Data analysis and visualization were performed using GraphPad Prism 8.0. Statistical significance was determined using the two-tailed Student’s *t*-test for paired comparisons. The threshold for statistical significance was set at *p* < 0.05.

## 5. Conclusions

**Figure 7:**
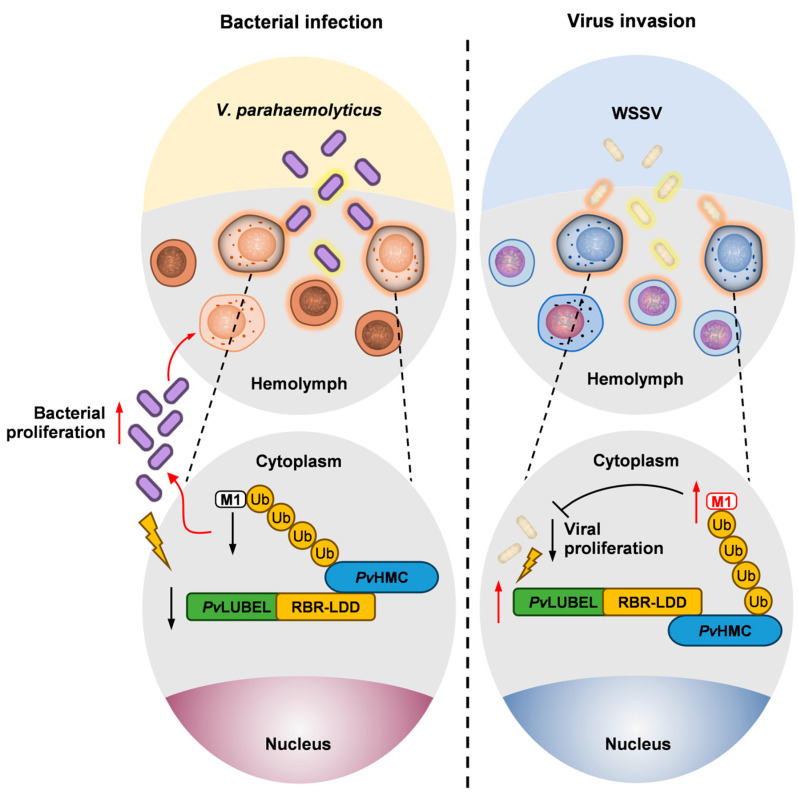
Proposed model of *Pv*HMC linear ubiquitination under pathogenic stress in *Penaeus vannamei* hemolymph and hemocytes. (**Left panel**): *V. parahaemolyticus* infection downregulates the E3 ubiquitin ligase *Pv*LUBEL, leading to reduced *Pv*HMC linear ubiquitination, weakened immune defense, and enhanced bacterial proliferation. (**Right panel**): WSSV infection upregulates *Pv*LUBEL expression, elevating *Pv*HMC linear ubiquitination, fortifying the shrimp immune response, and restricting viral replication.

Our study revealed the critical role of *Pv*LUBEL-mediated *Pv*HMC linear ubiquitination in the immune response of *Penaeus vannamei* against pathogens. Mechanistically, bacterial infections suppress *Pv*LUBEL expression and *Pv*HMC linear ubiquitination to evade immune clearance, while viral infections upregulate them to enhance antiviral immunity (Figure 7). Further investigation showed that inhibition of *Pv*LUBEL activity leads to reduce *Pv*HMC linear ubiquitination, severely impairing immune function, uncovering the indispensable role of *Pv*HMC linear ubiquitination in crustacean innate immunity.

## Data Availability

The data that support the findings of this study are available upon request from the corresponding author.

## Supplementary Materials

The following supporting information can be downloaded at https://www.mdpi.com/article/10.3390/ijms26115110/s1.

## References

[1] Swatek K.N., Komander D. (2016). Ubiquitin modifications. Cell Res..

[2] Buetow L., Huang D.T. (2016). Structural insights into the catalysis and regulation of E3 ubiquitin ligases. Nat. Rev. Mol. Cell Biol..

[3] Pao K.-C., Wood N.T., Knebel A., Rafie K., Stanley M., Mabbitt P.D., Sundaramoorthy R., Hofmann K., van Aalten D.M.F., Virdee S. (2018). Activity-based E3 ligase profiling uncovers an E3 ligase with esterification activity. Nature.

[4] Ikeda F. (2023). Protein and nonprotein targets of ubiquitin modification. Am. J. Physiol. Cell Physiol..

[5] Ciechanover A. (2015). The unravelling of the ubiquitin system. Nat. Rev. Mol. Cell Biol..

[6] Meyer H.-J., Rape M. (2014). Enhanced Protein Degradation by Branched Ubiquitin Chains. Cell.

[7] Fuseya Y., Fujita H., Kim M., Ohtake F., Nishide A., Sasaki K., Saeki Y., Tanaka K., Takahashi R., Iwai K. (2020). The HOIL-1L ligase modulates immune signalling and cell death via monoubiquitination of LUBAC. Nat. Cell Biol..

[8] Liu J., Wang Y., Gong Y., Fu T., Hu S., Zhou Z., Pan L. (2017). Structural Insights into SHARPIN-Mediated Activation of HOIP for the Linear Ubiquitin Chain Assembly. Cell Rep..

[9] Fuseya Y., Kadoba K., Liu X., Suetsugu H., Iwasaki T., Ohmura K., Sumida T., Kochi Y., Morinobu A., Terao C. (2024). Attenuation of HOIL-1L ligase activity promotes systemic autoimmune disorders by augmenting linear ubiquitin signaling. JCI Insight.

[10] Wang C., Gu C., Lv Y., Liu H., Wang Y., Zuo Y., Jiang G., Liu L., Liu J. (2024). AlphaFold2 assists in providing novel mechanistic insights into the interactions among the LUBAC subunits. Acta Biochim. Biophys. Sin..

[11] Wu Z., Berlemann L.A., Bader V., Sehr D.A., Dawin E., Covallero A., Meschede J., Angersbach L., Showkat C., Michaelis J.B. (2022). LUBAC assembles a ubiquitin signaling platform at mitochondria for signal amplification and transport of NF-κB to the nucleus. EMBO J..

[12] Klein T., Fung S.Y., Renner F., Blank M., Dufour A., Kang S., Bolger-Munro M., Scurll J.M., Priatel J.J., Schweigler P. (2015). The paracaspase MALT1 cleaves HOIL1 reducing linear ubiquitination by LUBAC to dampen lymphocyte NF-κB signalling. Nat. Commun..

[13] Belgnaoui S.M., Paz S., Samuel S., Goulet M.-L., Sun Q., Kikkert M., Iwai K., Dikic I., Hiscott J., Lin R. (2012). Linear Ubiquitination of NEMO Negatively Regulates the Interferon Antiviral Response through Disruption of the MAVS-TRAF3 Complex. Cell Host Microbe.

[14] Chen Y., Liu Y., Jiang K., Wen Z., Cao X., Wu S. (2023). Linear ubiquitination of LKB1 activates AMPK pathway to inhibit NLRP3 inflammasome response and reduce chondrocyte pyroptosis in osteoarthritis. J. Orthop. Transl..

[15] Asaoka T., Almagro J., Ehrhardt C., Tsai I., Schleiffer A., Deszcz L., Junttila S., Ringrose L., Mechtler K., Kavirayani A. (2016). Linear ubiquitination by LUBEL has a role in Drosophila heat stress response. EMBO Rep..

[16] Aalto A., Mohan A.K., Schwintzer L., Kupka S., Kietz C., Walczak H., Broemer M., Meinander A. (2019). M1-linked ubiquitination by LUBEL is required for inflammatory responses to oral infection in Drosophila. Cell Death Differ..

[17] Aalto A., Martínez-Chacón G., Kietz C., Tsyganova N., Kreutzer J., Kallio P., Broemer M., Meinander A. (2022). M1-linked ubiquitination facilitates NF-κB activation and survival during sterile inflammation. FEBS J..

[18] Ji R., Guan L., Hu Z., Cheng Y., Cai M., Zhao G., Zang J. (2024). A comprehensive review on hemocyanin from marine products: Structure, functions, its implications for the food industry and beyond. Int. J. Biol. Macromol..

[19] Li J., Zhao M., Zhang X., Zheng Z., Yao D., Yang S., Chen T., Zhang Y., Aweya J.J. (2024). The evolutionary adaptation of shrimp hemocyanin subtypes and the consequences on their structure and functions. Fish Shellfish Immunol..

[20] Coates C.J., Decker H. (2017). Immunological properties of oxygen-transport proteins: Hemoglobin, hemocyanin and hemerythrin. Cell. Mol. Life Sci..

[21] Sairi F., Gomes V.G., Dehghani F., Valtchev P. (2022). Lipoprotein-induced cell growth and hemocyanin biosynthesis in rhogocytes. Cell Tissue Res..

[22] Li Z.S., Ma S., Shan H.W., Wang T., Xiao W. (2019). Responses of hemocyanin and energy metabolism to acute nitrite stress in juveniles of the shrimp *Litopenaeus vannamei*. Ecotoxicol. Environ. Saf..

[23] Li C., Wang F., Aweya J.J., Yao D., Zheng Z., Huang H., Li S., Zhang Y. (2018). Trypsin of *Litopenaeus vannamei* is required for the generation of hemocyanin-derived peptides. Dev. Comp. Immunol..

[24] Zhao X., Qiao J., Zhang P., Zhang Z., Aweya J.J., Chen X., Zhao Y., Zhang Y. (2021). Protein Diversity and Immune Specificity of Hemocyanin From Shrimp *Litopenaeus vannamei*. Front. Immunol..

[25] Zhang Z., Li R., Aweya J.J., Wang F., Zhong M., Zhang Y. (2019). Identification and characterization of glycosylation sites on *Litopenaeus vannamei* hemocyanin. FEBS Lett..

[26] Feng Q., Aweya J.J., Huang Y.-Q., Zhang P., Wang F., Yao D.-F., Zheng Z.-H., Li E.-M., Zhang Y.-L. (2023). Dephosphorylation of T517 on Hemocyanin Is Required for Antibacterial Activity in *Penaeus vannamei*. J. Immunol..

[27] Nie J., Aweya J.J., Yu Z., Zhou H., Wang F., Yao D., Zheng Z., Li S., Ma H., Zhang Y. (2022). Deacetylation of K481 and K484 on Penaeid Shrimp Hemocyanin Is Critical for Antibacterial Activity. J. Immunol..

[28] Kumar M., Chadha N.K., Prakash S., Pavan-Kumar A., Harikrishna V., Gireesh-Babu P., Krishna G. (2024). Salinity, stocking density, and their interactive effects on growth performance and physiological parameters of white-leg shrimp, *Penaeus vannamei* (Boone, 1931), reared in inland ground saline water. Aquac. Int..

[29] Joffre O.M., Poortvliet P.M., Klerkx L. (2018). Are shrimp farmers actual gamblers? An analysis of risk perception and risk management behaviors among shrimp farmers in the Mekong Delta. Aquaculture.

[30] Andrade T.P.D., Cruz-Flores R., Mai H.N., Dhar A.K. (2022). Novel infectious myonecrosis virus (IMNV) variant is associated with recent disease outbreaks in *Penaeus vannamei* shrimp in Brazil. Aquaculture.

[31] Phanse Y., Puttamreddy S., Loy D., Ramirez J.V., Ross K.A., Alvarez-Castro I., Mogler M., Broderick S., Rajan K., Narasimhan B. (2022). RNA Nanovaccine Protects against White Spot Syndrome Virus in Shrimp. Vaccines.

[32] Mai H.N., Aranguren Caro L.F., Cruz-Flores R., Dhar A.K. (2021). Development of a Recombinase Polymerase Amplification (RPA) assay for acute hepatopancreatic necrosis disease (AHPND) detection in Pacific white shrimp (*Penaeus vannamei*). Mol. Cell. Probes.

[33] Reyes G., Betancourt I., Andrade B., Panchana F., Román R., Sorroza L., Trujillo L.E., Bayot B. (2022). Microbiome of *Penaeus vannamei* Larvae and Potential Biomarkers Associated with High and Low Survival in Shrimp Hatchery Tanks Affected by Acute Hepatopancreatic Necrosis Disease. Front. Microbiol..

[34] Keusekotten K., Elliott P.R., Glockner L., Fiil B.K., Damgaard R.B., Kulathu Y., Wauer T., Hospenthal M.K., Gyrd-Hansen M., Krappmann D. (2013). OTULIN Antagonizes LUBAC Signaling by Specifically Hydrolyzing Met1-Linked Polyubiquitin. Cell.

[35] Stieglitz B., Rana R.R., Koliopoulos M.G., Morris-Davies A.C., Schaeffer V., Christodoulou E., Howell S., Brown N.R., Đikić I., Rittinger K. (2013). Structural basis for ligase-specific conjugation of linear ubiquitin chains by HOIP. Nature.

[36] Katsuya K., Hori Y., Oikawa D., Yamamoto T., Umetani K., Urashima T., Kinoshita T., Ayukawa K., Tokunaga F., Tamaru M. (2018). High-Throughput Screening for Linear Ubiquitin Chain Assembly Complex (LUBAC) Selective Inhibitors Using Homogenous Time-Resolved Fluorescence (HTRF)-Based Assay System. SLAS Discov..

[37] Katsuya K., Oikawa D., Iio K., Obika S., Hori Y., Urashima T., Ayukawa K., Tokunaga F. (2019). Small-molecule inhibitors of linear ubiquitin chain assembly complex (LUBAC), HOIPINs, suppress NF-κB signaling. Biochem. Biophys. Res. Commun..

[38] Yang P., Aweya J.J., Yao D., Wang F., Lun J., Hong Y., Sun K., Zhang Y. (2020). The krüppel-like factor of *Penaeus vannamei* negatively regulates transcription of the small subunit hemocyanin gene as part of shrimp immune response. Fish Shellfish Immunol..

[39] Zhao M., Aweya J.J., Feng Q., Zheng Z., Yao D., Zhao Y., Chen X., Zhang Y. (2022). Ammonia stress affects the structure and function of hemocyanin in *Penaeus vannamei*. Ecotoxicol. Environ. Saf..

[40] Wang Z., Aweya J.J., Yao D., Zheng Z., Wang C., Zhao Y., Li S., Zhang Y. (2022). Taurine metabolism is modulated in Vibrio-infected *Penaeus vannamei* to shape shrimp antibacterial response and survival. Microbiome.

[41] Rubio T., Oyanedel D., Labreuche Y., Toulza E., Luo X., Bruto M., Chaparro C., Torres M., de Lorgeril J., Haffner P. (2019). Species-specific mechanisms of cytotoxicity toward immune cells determine the successful outcome of *Vibrio* infections. Proc. Natl. Acad. Sci. USA.

[42] Zhang Z., Aweya J.J., Yao D., Zheng Z., Tran N.T., Li S., Zhang Y. (2021). Ubiquitination as an Important Host-Immune Response Strategy in Penaeid Shrimp: Inferences From Other Species. Front. Immunol..

[43] Zhao M., Zheng Z., Wang C., Yao D., Lin Z., Zhao Y., Chen X., Li S., Aweya J.J., Zhang Y. (2023). Penaeid shrimp counteract high ammonia stress by generating and using functional peptides from hemocyanin, such as HMCs27. Sci. Total Environ..

[44] Chen D.-D., Jiang J.-Y., Lu L.-F., Zhang C., Zhou X.-Y., Li Z.-C., Zhou Y., Li S. (2021). Zebrafish Uba1 Degrades IRF3 through K48-Linked Ubiquitination to Inhibit IFN Production. J. Immunol..

[45] Samant R.S., Livingston C.M., Sontag E.M., Frydman J. (2018). Distinct proteostasis circuits cooperate in nuclear and cytoplasmic protein quality control. Nature.

[46] Sheng X., Xia Z., Yang H., Hu R. (2023). The ubiquitin codes in cellular stress responses. Protein Cell.

[47] Wu X., Zhou L., Ye C., Zha Z., Li C., Feng C., Zhang Y., Jin Q., Pan J. (2023). Destruction of self-derived PAMP via T3SS2 effector VopY to subvert PAMP-triggered immunity mediates *Vibrio parahaemolyticus* pathogenicity. Cell Rep..

[48] Tran N.T., Liang H., Li J., Deng T., Bakky M.A.H., Zhang M., Li S. (2023). Cellular responses in crustaceans under white spot syndrome virus infection. Fish Shellfish Immunol..

[49] Shariq M., Quadir N., Alam A., Zarin S., Sheikh J.A., Sharma N., Samal J., Ahmad U., Kumari I., Hasnain S.E. (2022). The exploitation of host autophagy and ubiquitin machinery by Mycobacterium tuberculosis in shaping immune responses and host defense during infection. Autophagy.

[50] Noad J., von der Malsburg A., Pathe C., Michel M.A., Komander D., Randow F. (2017). LUBAC-synthesized linear ubiquitin chains restrict cytosol-invading bacteria by activating autophagy and NF-κB. Nat. Microbiol..

[51] Lechtenberg B.C., Rajput A., Sanishvili R., Dobaczewska M.K., Ware C.F., Mace P.D., Riedl S.J. (2016). Structure of a HOIP/E2~ubiquitin complex reveals RBR E3 ligase mechanism and regulation. Nature.

[52] Mengal K., Kor G., Siino V., Buřič M., Kozák P., Levander F., Niksirat H. (2023). Quantification of proteomic profile changes in the hemolymph of crayfish during in vitro coagulation. Dev. Comp. Immunol..

[53] Yan Y., Hillyer J.F. (2020). The immune and circulatory systems are functionally integrated across insect evolution. Sci. Adv..

[54] Oikawa D., Sato Y., Ohtake F., Komakura K., Hanada K., Sugawara K., Terawaki S., Mizukami Y., Phuong H.T., Iio K. (2020). Molecular bases for HOIPINs-mediated inhibition of LUBAC and innate immune responses. Commun. Biol..

[55] Fennell L.M., Diaz C.G., Deszcz L., Kavirayani A., Hoffmann D., Yanagitani K., Schleiffer A., Mechtler K., Hagelkruys A., Penninger J. (2020). Site-specific ubiquitination of the E3 ligase HOIP regulates apoptosis and immune signaling. EMBO J..

[56] Xu G., Wu Y., Xiao T., Qi F., Fan L., Zhang S., Zhou J., He Y., Gao X., Zeng H. (2022). Multiomics approach reveals the ubiquitination-specific processes hijacked by SARS-CoV-2. Signal Transduct. Target. Ther..

[57] Yao D., Wang Z., Wei M., Zhao X., Aweya J.J., Zhong M., Li S., Zhang Y. (2019). Analysis of *Litopenaeus vannamei* hemocyanin interacting proteins reveals its role in hemolymph clotting. J. Proteom..

[58] Varadi M., Bertoni D., Magana P., Paramval U., Pidruchna I., Radhakrishnan M., Tsenkov M., Nair S., Mirdita M., Yeo J. (2023). AlphaFold Protein Structure Database in 2024: Providing structure coverage for over 214 million protein sequences. Nucleic Acids Res..

[59] E3 Ubiquitin-Protein Ligase Lubel [*Penaeus vannamei*]. https://www.ncbi.nlm.nih.gov/protein/XP_069991990.1/.

[60] Letunic I., Khedkar S., Bork P. (2020). SMART: Recent updates, new developments and status in 2020. Nucleic Acids Res..

[61] Forli S., Huey R., Pique M.E., Sanner M.F., Goodsell D.S., Olson A.J. (2016). Computational protein–ligand docking and virtual drug screening with the AutoDock suite. Nat. Protoc..

[62] DeLano W.L. (2015). The PyMOL Molecular Graphics System.

[63] Schroeder A.B., Dobson E.T.A., Rueden C.T., Tomancak P., Jug F., Eliceiri K.W. (2021). The ImageJ ecosystem: Open-source software for image visualization, processing, and analysis. Protein Sci..

