# Supplementary material for: Linear Ubiquitination of Hemocyanin Mediated by LUBEL Regulates Innate Immunity in Penaeus vannamei
Source: Int J Mol Sci. 2025 May 26;26(11):5110. doi: 10.3390/ijms26115110 (PMC12154395; doi:10.3390/ijms26115110)
Supplement: Supplementary file 1 [file ijms-26-05110-s001.zip › ijms-3594889-supplementary.pdf]

Supplementary Materials

Linear Ubiquitination of Hemocyanin Mediated by LUBEL Regulates Innate Immunity in *Penaeus vannamei*

Xiaojun Zhang, Hanfeng Zhang, Yueling Zhang, Zhongyang Lin

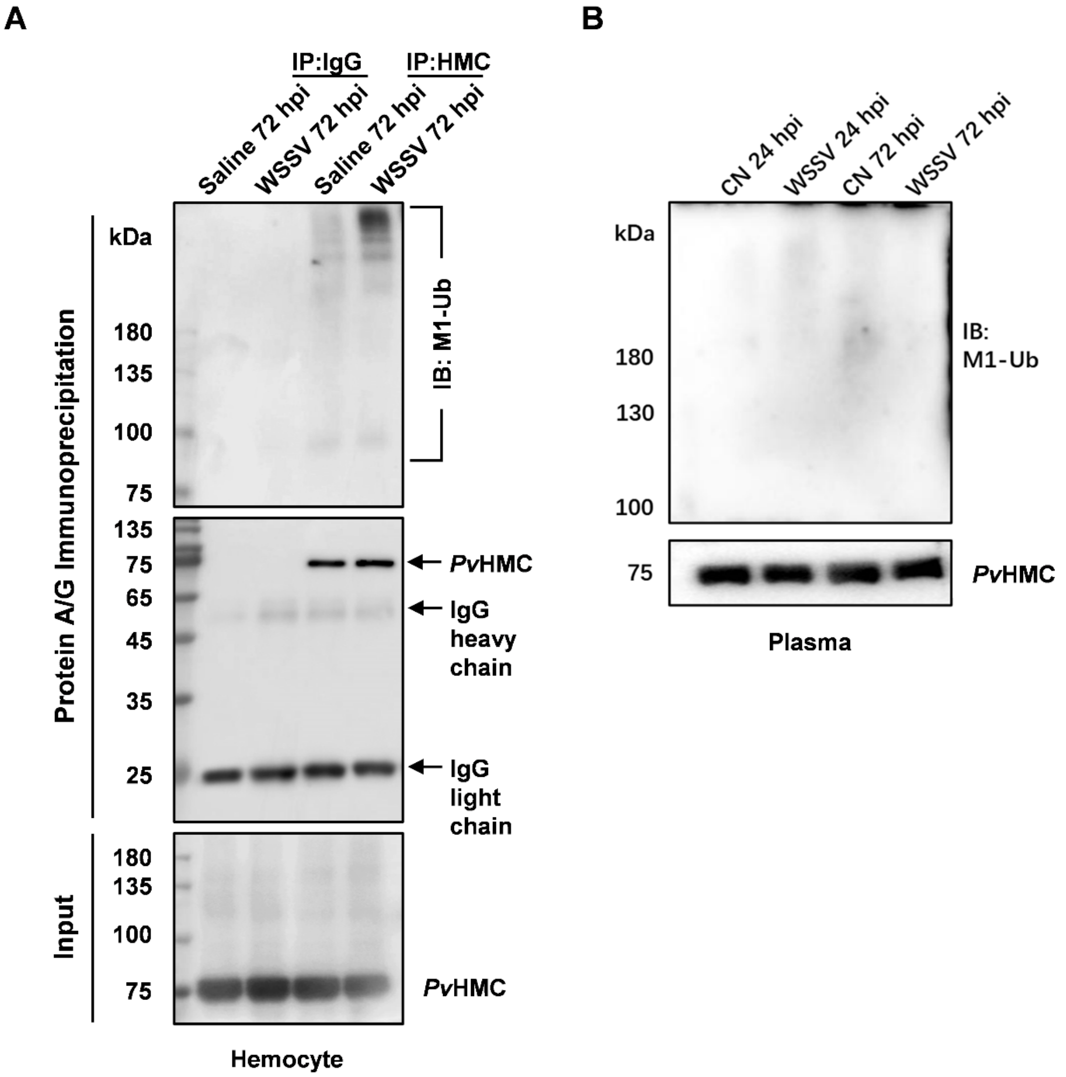

Figure S1. Identification of linear ubiquitination of *Pv*HMC. (**A**) Western blotting analysis of the specificity of linear ubiquitinated antibodies for *Pv*HMC after immunoprecipitation (IP), with IgG as a control, untreated or WSSV-stimulated for 72 h. (**B**) Western blotting analysis of *Pv*HMC linear ubiquitination in plasma (without IP), untreated or WSSV-stimulated for 24 and 72 h.

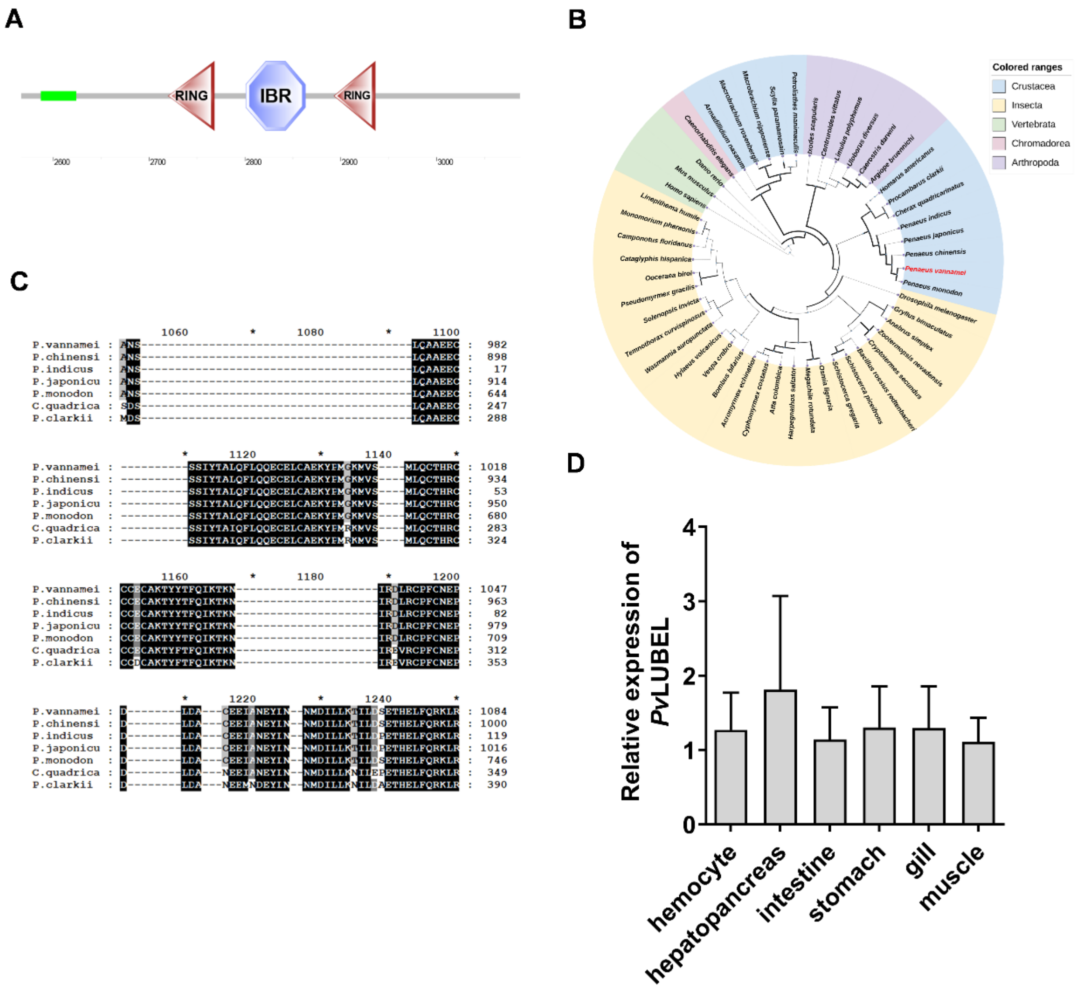

Figure S2. Sequence bioinformatics and phylogenetic analysis of *Pv*LUBEL. (**A**) Domain prediction analysis of *Pv*LUBEL, only partial regions (2600-3000 aa) are shown in the figure, including RBR domain and a coiled coil region (green). (**B**) The phylogenetic analysis of *Pv*LUBEL-RBR domain showed relationships between different invertebrates based on coding *Pv*LUBEL-RBR sequence comparisons. The phylogenetic tree was generated using MEGA 11.0 with maximum likelihood method. The *Penaeus vannamei* subfield was marked in coloured. (**C**) Amino acid sequence deduced from cDNA of *Pv*LUBEL. The RBR domain was emphasized in black. (**D**) Tissue-specific expression of *Pv*LUBEL in the hepatopancreas, hemocytes, stomach, gills, intestine and muscle.

Table S1. Nucleotide sequences of the primers for Real-time PCR and RNAi

Primers	Sequence (5'-3')
**Real-time PCR primers**	
**q*Pv*LUBEL-F**	**ACGCAGTGCAAGTACGAGTT**
**q*Pv*LUBEL-R**	**GTCGTAGGTGATTCCCGCTT**
**q*Pv*EF-1α-F**	**TATGCTCCTTTTGGACGTTTTGC**
**q*Pv*EF-1α-R**	**CCTTTTCTGCGGCCTTGGTAG**
**q*Vibrio*-F**	**GGCGTAAAGCGCATGCAGGT**
**q*Vibrio*-R**	**GAAATTCTACCCCCCTCTACAG**
**qVP28-F**	**AAACCTCCGCATTCCTGTGA**
**qVP28-R**	**TCCGCATCTTCTTCCTTCAT**
**dsRNA primers**	
**dsLUBEL-F**	**GTGAGCCGAATCCTTCCCTC**
**dsLUBEL-R**	**TCATCGTTGTGGGCCTTCAC**
**dsLUBEL-T7F**	**GGATCCTAATACGACTCACTATAGG**
	**GTGAGCCGAATCCTTCCCTC**
**dsLUBEL-T7R**	**GGATCCTAATACGACTCACTATAGG**
	**TCATCGTTGTGGGCCTTCAC**
**dsEGFP-T7-F**	**GGATCCTAATACGACTCACTATAGG**
	**CGTAAACGGCCACAAGTT**
**dsEGFP-R**	**TTCACCTTGATGCCGTTC**
**dsEGFP-F**	**CGTAAACGGCCACAAGTT**
**dsEGFP-T7-R**	**GGATCCTAATACGACTCACTATAGG**
	**TTCACCTTGATGCCGTTC**
